# Tn5-Labeled DNA-FISH: An Optimized Probe Preparation Method for Probing Genome Architecture

**DOI:** 10.3390/ijms26052224

**Published:** 2025-02-28

**Authors:** Yang Yang, Gengzhan Chen, Tong Gao, Duo Ning, Yuqing Deng, Zhongyuan (Simon) Tian, Meizhen Zheng

**Affiliations:** 1 Shenzhen Key Laboratory of Gene Regulation and Systems Biology, School of Life Sciences, Southern University of Science and Technology, Shenzhen 518055, China; 12131322@mail.sustech.edu.cn (Y.Y.); 12031138@mail.sustech.edu.cn (D.N.); 2 Department of Systems Biology, School of Life Sciences, Southern University of Science and Technology, Shenzhen 518055, China; 12233150@mail.sustech.edu.cn (G.C.); 12331396@mail.sustech.edu.cn (T.G.); 12233113@mail.sustech.edu.cn (Y.D.)

**Keywords:** Tn5 transposome, DNA-FISH, probe preparation, chromatin spatial conformation, gene interactions

## Abstract

Three-dimensional genome organization reveals that gene regulatory elements, which are linearly distant on the genome, can spatially interact with target genes to regulate their expression. DNA fluorescence in situ hybridization (DNA-FISH) is an efficient method for studying the spatial proximity of genomic loci. In this study, we developed an optimized Tn5 transposome-based DNA-FISH method, termed Tn5-labeled DNA-FISH. This approach amplifies the target region and uses a self-assembled Tn5 transposome to simultaneously fragment the DNA into ~100 bp segments and label it with fluorescent oligonucleotides in a single step. This method enables the preparation of probes for regions as small as 4 kb and visualizes both endogenous and exogenous genomic loci at kb resolution. Tn5-labeled DNA-FISH provides a streamlined and cost-effective tool for probe generation, facilitating the investigation of chromatin spatial conformations, gene interactions, and genome architecture.

## 1. Introduction

The genome of eukaryotic cells is highly compacted within the nucleus, forming a complex three-dimensional chromatin spatial conformation that is essential for gene regulation and biological processes [1]. Regulatory elements that are linearly distant on the genome can spatially interact with target genes, influencing their expression [2]. These spatial interactions provide critical insights into how non-coding variants affect gene regulation, offering powerful evidence for understanding the mechanisms underlying gene expression changes [3]. Genome-wide sequencing-based technologies, such as high- throughput chromosome conformation capture (Hi-C) [4] and chromatin interaction analysis with paired-end tag (ChIA-PET) [5], have mapped long-range chromatin interactions between regulatory elements and target genes. However, DNA-FISH remains an indispensable tool for validating specific spatial interactions between loci due to its ability to directly visualize chromatin interactions and gene positioning [6,7].

Traditional DNA-FISH methods rely on large DNA templates, such as BAC clones, to prepare probes. These BAC-based probes, typically 100–300 kb in size [8], are generated through labor-intensive processes involving plasmid transfection, DNA extraction, enzymatic fragmentation, and fluorescent labeling [9]. While effective, these probes suffer from low resolution, as chromatin interactions often occur within 100 kb [10], and their specificity is compromised by repetitive sequences within BAC clones. These limitations hinder the study of small-scale chromatin structures, such as chromatin loops and topologically associated domains (TADs), which are often at the kilobase (kb) scale [11].

To overcome these challenges, oligo-based methods, such as Oligopaint [12] and MERFISH [13], have gained prominence in recent decades, offering significant advantages in detecting chromatin interactions. A new gene-based physical mapping approach can avoid repetitive DNA by utilizing complementary DNA as a template for generating probes [14]. Inspired by these innovations, a Tn5 transposome-based [15] DNA-FISH method has been previously described [16], along with data analyzation techniques [17]. In this study, we optimized a Tn5 transposome-based DNA-FISH method, termed Tn5-labeled DNA-FISH, which builds upon these studies by streamlining the probe preparation process. This technique directly amplifies the target loci from genomic DNA at the kb scale as probe templates. Using a self-assembled Tn5 transposome with pre-labeled fluorescent oligonucleotides, the method simultaneously fragments and labels DNA in a single step. This approach eliminates the need for an additional round of PCR required in previous methods, where labeled primers were used to add the fluorescent label to the fragmented DNA. As a result, our method simplifies probe preparation while avoiding the issue of fluorescent primer contamination.

In this paper, Tn5-labeled DNA-FISH has been successfully applied to visualize both endogenous and exogenous genomic loci at a kb resolution. Furthermore, we have utilized this method to analyze genomic loci and demonstrate chromatin loops in the region using high-resolution microscopy [18]. Thus, this method provides a robust and practical tool for high-resolution chromatin interaction studies. Here, we present a detailed step-by-step protocol for Tn5-labeled DNA-FISH, from probe preparation and hybridization to imaging.

## 2. Experimental Design

**Figure 1:**
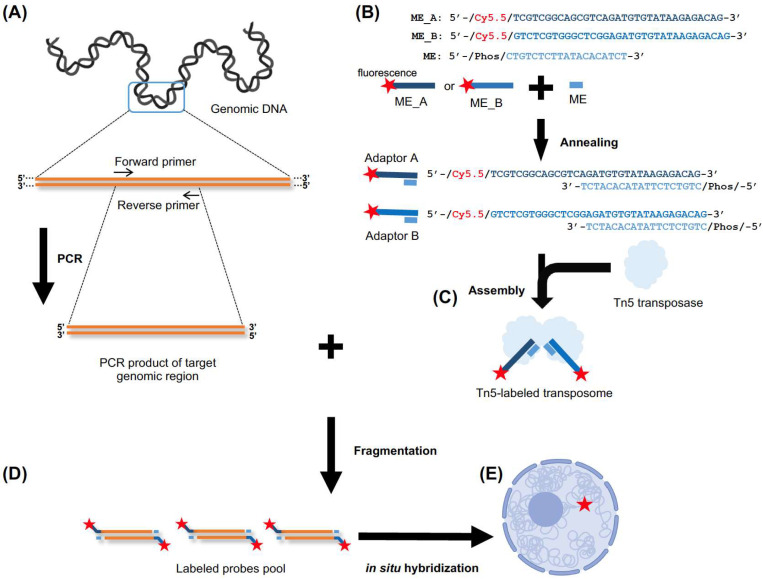
The overall workflow of Tn5-labeled DNA-FISH is illustrated. (**A**) The target genomic region is amplified by PCR with specific primers. (**B**) Fluorescently labeled adaptors (ME) are synthesized and annealed, with the Cy5.5 fluorophore indicated by the red star. ME_A (dark blue) or ME_B (blue) is annealed with ME (light blue) to form Adaptor A and Adaptor B, respectively. The blue bars correspond to the DNA sequences of the oligonucleotides. (**C**) The fluorescently labeled adaptors are assembled with Tn5 transposase to form the Tn5-labeled transposome. (**D**) The Tn5-labeled transposome performs fragmentation on the target genomic DNA, simultaneously fragmenting the DNA and integrating the fluorescently labeled adaptors to generate labeled probes. (**E**) The labeled probes are then used for DNA-FISH experiments.

This protocol outlines the Tn5-labeled DNA-FISH technique for detecting endogenous genomic loci in *Drosophila* cells and exogenous Epstein–Barr virus (EBV) genomes in human cells. The general procedure is illustrated in Figure 1. In brief, genomic DNA was extracted, and the target genomic region was amplified by PCR using specific primers to generate the probe template. Meanwhile, Tn5-labeled transposome was assembled by fluorescently labeled adaptors and Tn5 transposase. Then, labeled probes were generated by tagmentation of Tn5-labeled transposome on the amplified target genomic DNA. This process simultaneously fragments the DNA and integrates the fluorescently labeled adaptors into the DNA fragments. Finally, the labeled probes were used for DNA-FISH experiments.

## 3. Materials

### 3.1. Reagents and Consumables

- 0.1 M dithiothreitol (DTT) (Thermo Fisher Scientific, Waltham, MA, USA, #707265ML).
- 0.5 M EDTA (Invitrogen, Waltham, MA, USA, #AM9260G).
- 1 M citric acid (pH 6.0) (Macgene, Beijing, China, #MC009).
- 1 M magnesium acetate (Sigma-Aldrich, Burlington, VT, USA, #63052-100ML).
- 1 M Tris-HCl pH 7.0 (Invitrogen, Waltham, MA, USA, #AM9851).
- 1 M Tris-HCl pH 8.0 (Invitrogen, Waltham, MA, USA, #AM9856).
- 1.5 mL DNA LoBind Tubes (Eppendorf, Hamburg, Germany, #30108051).
- 10 mM dATP (Invitrogen, Waltham, MA, USA, #18252015).
- 10% SDS (*w*/*v*) (Invitrogen, Waltham, MA, USA, #AM9822).
- 10× TBE Buffer (Invitrogen, Waltham, MA, USA, #AM9863).
- 11.6–12.0 M HCl (MERCK, Darmstadt, Germany, #1.00317.2508).
- 15 cm plate (JET Biofil, Shanghai, China, #CAD010150).
- 20× SSC Buffer (Invitrogen, Waltham, MA, USA, #AM9770).
- 200 μL PCR tubes (Sangon Biotech, Shanghai, China, #F611541-0010).
- 2 mL DNA LoBind tubes (Eppendorf, Hamburg, Germany, #22431048).
- 4% paraformaldehyde (PFA) (BosterBio, Wuhan, China, #AR1068).
- 4–20% Novex TBE Gels (Invitrogen, Waltham, MA, USA, #EC6225BOX).
- 5 M NaCl (Invitrogen, Waltham, MA, USA, #AM9759).
- 5 M potassium acetate (Sigma-Aldrich, Burlington, VT, USA, #95843-100ML-F).
- 50× Denhardt’s solution (Sangon Biotech, Shanghai, China, #B548209-0050).
- 6-well plate (JET Biofil, Shanghai, China, #TCP010006).
- Buffer EB (Qiagen, Hilden, Germany, #19086).
- Coverslips (Thorlabs, Newton, NJ, USA, #CG15XH).
- Dextran sulfate (Sigma-Aldrich, Burlington, VT, USA, #D8906).
- DNA Clean & Concentrator-5 (Zymo Research, Irvine, CA, USA, #D4013).
- Dulbecco’s Phosphate-Buffered Saline (DPBS) (Gibco, Waltham, MA, USA, #14190250).
- Ethyl alcohol (Sigma-Aldrich, Burlington, VT, USA, #E7023-500ML).
- Fetal bovine serum (ExCell Bio, Shanghai, China, #FSP500).
- Formamide (deionized) (Invitrogen, Waltham, MA, USA, #AM9342).
- Glycerol (Sigma-Aldrich, Burlington, VT, USA, #G2025-100mL).
- Heparin (Sigma-Aldrich, Burlington, VT, USA, #H3149-10KU).
- N, N-dimethylformamide (DMF) (Sigma-Aldrich, Burlington, VT, USA, #227056-100ML).
- N1 Cartridge (High Sensitivity Cartridge) (Bioptic, Beijing, China, #C105105).
- Nuclease-Free Water (not DEPC-treated) (Invitrogen, Waltham, MA, USA, #AM9932).
- Nuclease-Free Water (not DEPC-treated) (Invitrogen, Waltham, MA, USA, #AM9937).
- Oligos (GENEWIZ, Suzhou, China).
- Penicillin/streptomycin (Gibco, Waltham, MA, USA, #15140122).
- Phanta Max Super-Fidelity DNA Polymerase (Vazyme, Nanjing, China, #P505-d1).
- Poly-L-Lysine (Sigma-Aldrich, Burlington, VT, USA, #P4707).
- Proteinase K Solution (Invitrogen, Waltham, MA, USA, #AM2548).
- Qubit dsDNA HS Assay Kit (Invitrogen, Waltham, MA, USA, #Q32851).
- RNase A (10 mg/mL) (Thermo Fisher Scientific, Waltham, MA, USA, #EN0531).
- RNase-free 15 mL tubes (Invitrogen, Waltham, MA, USA, #AM12500).
- RNase-free 50 mL tubes, (Invitrogen, Waltham, MA, USA, #AM12502).
- RPMI 1640 Medium (Gibco, Waltham, MA, USA, #A1049101).
- S3 Cartridge (Kilo Base Cartridge) (Bioptic, Beijing, China, #C106106).
- Safe green (Biosharp, Hefei, China, #BS356A).
- Schneider’s Drosophila Medium (Gibco, Waltham, MA, USA, #21720024).
- TE Buffer pH 8.0 (Invitrogen, Waltham, MA, USA, #AM9849).
- TIANamp Genomic DNA Kit (TIANGEN, Beijing, China, #DP304).
- Tn5 transposase (BGI, Shenzhen, China, #LS-EZ-E-00009O).
- Triton X-100 (Acros Organics, Geel, Belgium, #327371000-100mL).
- Tween 20 (Sigma-Aldrich, Burlington, VT, USA, #P1379-100ML).
- Ultra Low Range DNA Ladder (Invitrogen, Waltham, MA, USA, #10597012).

### 3.2. Equipment

- C1000 Touch Thermal Cycler (Bio-Rad, Hercules, CA, USA, #1851148).
- Eppendorf 5424R Centrifuge (Eppendorf, Hamburg, Germany, #5404F1621754).
- Eppendorf 5425 Centrifuge (Eppendorf, Hamburg, Germany, #5405000204).
- Eppendorf 5810R Centrifuge (Eppendorf, Hamburg, Germany, #22628180).
- Eppendorf Thermomixer C (Eppendorf, Hamburg, Germany, #5382000023).
- Gel imaging system (Tanon, Shanghai, China, #Tanon 3500R).
- Intelli-mixer RM-2L (ELMI, Riga, Latvia, #RM-2L).
- Magnetic stirrer (CRYSTAL, Shanghai, China, #MS2-P1H).
- Nikon Eclipse TS2 Microscope (Nikon, Tokyo, Japan, #Eclipse TS2).
- Qsep100 (Bioptic, Beijing, China).
- Qubit 4.0 Fluorometer (Invitrogen, Waltham, MA, USA, #Q33226).
- Refrigerator (Haier, Qingdao, China, #DW-25L262).
- Thermo Scientific Heratherm IMH180 Advanced Microbiological Incubator (Thermo Fisher Scientific, Waltham, MA, USA, #IMH180).
- Thermo Scientific IMP180 Heratherm Refrigerated Incubators (Thermo Fisher Scientific, Waltham, MA, USA, #IMP180).
- Thermo Scientific Heracell VIOS 160i CO_2_ Incubator (Thermo Fisher Scientific, Waltham, MA, USA, #51033549).
- Ultra-Low Temperature Freezers (Thermo Fisher Scientific, Waltham, MA, USA, #995).

### 3.3. Oligo Sequences

The oligos used in this study are listed in Table 1.

### 3.4. Solutions and Buffers

- 1× TNE buffer: Add 9.798 mL of Nuclease-Free Water (Invitrogen, Waltham, MA, USA) to a 15 mL tube first and then sequentially add 100 µL of 1 M Tris-HCl pH 8.0 (Invitrogen, Waltham, MA, USA), 100 µL of 5 M NaCl (Invitrogen, Waltham, MA, USA), and 2 µL of 0.5 M EDTA (Invitrogen, Waltham, MA, USA); mix the solution well. The buffer can be stored at room temperature for up to 6 months.
- 20% Triton X-100: Add 40 mL of Nuclease-Free Water (Invitrogen, Waltham, MA, USA) to a 50 mL tube first and then slowly add 10 mL of Triton X-100 (Acros Organics, Geel, Belgium); mix the solution well by rotation overnight. The buffer can be stored at room temperature for up to 6 months. CRITICAL: Triton X-100 is highly viscous; aspirate and dispense it slowly using pipette tips. Pipette up and down several times to ensure all Triton X-100 adhering to the tip walls is recovered. Protect the buffer from light.
- Coupling buffer: Add 2.174 mL of Nuclease-Free Water (Invitrogen, Waltham, MA, USA) to a 15 mL tube first and then sequentially add 125 µL of 1 M Tris-HCl pH 7.0 (Invitrogen, Waltham, MA, USA), 125 µL of 1 M Tris-HCl pH 8.0 (Invitrogen, Waltham, MA, USA), 1 µL of 0.5 M EDTA (Invitrogen, Waltham, MA, USA), 50 µL of 0.1 M DTT (Thermo Fisher Scientific, Waltham, MA, USA), 25 µL of 20% Triton X-100, and 2.5 mL of glycerol (Sigma-Aldrich, Burlington, VT, USA); mix the solution well. The buffer can be stored at −20 °C for up to 1 year.
- 4× THS TD buffer: Add 676 µL of Nuclease-Free Water (Invitrogen, Waltham, MA, USA) to a 15 mL tube first and then sequentially add 660 µL of 1 M Tris-HCl pH 8.0 (Invitrogen, Waltham, MA, USA), 264 µL of 5 M potassium acetate (Sigma-Aldrich, Burlington, VT, USA), 200 µL of 1 M magnesium acetate (Sigma-Aldrich, Burlington, VT, USA), and 3.2 mL of DMF (Sigma-Aldrich, Burlington, VT, USA); mix the solution well. The buffer can be stored at −20 °C for up to 1 year. CRITICAL: manipulate DMF in a chemical hood.
- ChIP Elution buffer: Add 8.3 mL of Nuclease-Free Water (Invitrogen, Waltham, MA, USA) to a 15 mL tube first and then sequentially add 250 µL of 1 M Tris-HCl pH 7.0 (Invitrogen, Waltham, MA, USA), 250 µL of 1 M Tris-HCl pH 8.0 (Invitrogen, Waltham, MA, USA), 200 µL of 0.5 M EDTA (Invitrogen, Waltham, MA, USA), and 1 mL of 10% SDS (*w*/*v*) (Invitrogen, Waltham, MA, USA); mix the solution well. The buffer can be stored at room temperature for up to 6 months.
- 0.5% Triton X-100/DPBS: Add 1.95 mL of DPBS (Gibco, Waltham, MA, USA) to a 2 mL tube first and then add 50 µL of 20% Triton X-100; mix the solution well. The buffer can be stored at room temperature for up to one week.
- 100 µg/mL RNase A/DPBS: Add 1.98 mL of DPBS (Gibco, Waltham, MA, USA) to a 2 mL tube first and then add 20 µL of RNase A (10 mg/mL) (Thermo Fisher Scientific, Waltham, MA, USA); mix the solution well. CRITICAL: prepare the buffer freshly.
- 20% glycerol/DPBS: Add 1.6 mL of DPBS (Gibco, Waltham, MA, USA) to a 2 mL tube first and then add 400 µL of glycerol (Sigma-Aldrich, Burlington, VT, USA); mix the solution well. The buffer can be stored at room temperature for up to 6 months. CRITICAL: Glycerol is highly viscous; aspirate and dispense it slowly using pipette tips. Pipette up and down several times to ensure all glycerol adhering to the tip walls is recovered.
- 1 M HCl: Add 1.983 mL of Nuclease-Free Water (Invitrogen, Waltham, MA, USA) to a 2 mL tube first and then slowly add 17 µL of 11.6–12.0 M HCl (MERCK, Darmstadt, Germany); mix the solution well. CRITICAL: prepare the buffer freshly and carefully and manipulate in a chemical hood.
- 2× SSC: Add 45 mL of Nuclease-Free Water (Invitrogen, Waltham, MA, USA) to a 50 mL tube first and then add 5 mL of 20× SSC (Invitrogen, Waltham, MA, USA); mix the solution well. The buffer can be stored at room temperature for up to one week.
- 2× SSC/50% formamide: Add 2 mL of Nuclease-Free Water (Invitrogen, Waltham, MA, USA) to a 15 mL tube first and then add 500 µL of 20× SSC (Invitrogen, Waltham, MA, USA); mix the solution well. The stock buffer can be stored at room temperature. Add 2.5 mL of formamide (Invitrogen, Waltham, MA, USA) before use, mix the solution well. CRITICAL: Formamide is stored at 4 °C; pre-warm it to room temperature before taking. Add formamide before use. Manipulate formamide in a chemical hood.
- 10% Tween 20: Add 90 µL of Nuclease-Free Water (Invitrogen, Waltham, MA, USA) to a 1.5 mL tube first and then add 10 µL of Tween 20 (Sigma-Aldrich, Burlington, VT, USA); mix the solution well. The buffer can be stored at room temperature for up to 6 months. CRITICAL: Tween 20 is highly viscous; aspirate and dispense it slowly using pipette tips. Pipette up and down several times to ensure all Tween 20 adhering to the tip walls is recovered.
- Hybridization buffer: Add 5 µL of Nuclease-Free Water (Invitrogen, Waltham, MA, USA) to a 1.5 mL tube first and then sequentially add 125 µL of 20× SSC (Invitrogen, Waltham, MA, USA), 4.5 µL of 1 M citric acid (pH 6.0) (Macgene, Beijing, China), 250 µL of formamide (Invitrogen, Waltham, MA, USA), 5 µL of 10% Tween 20 (Sigma-Aldrich, Burlington, VT, USA), 0.5 µL of 50 mg/mL heparin (Sigma-Aldrich, Burlington, VT, USA), 10 µL of 50× Denhardt’s solution (Sangon Biotech, Shanghai, China), and 100 µL of 50% dextran sulfate (Sigma-Aldrich, Burlington, VT, USA); mix the solution well. CRITICAL: Prepare the buffer freshly. Formamide is stored at 4 °C; pre-warm it to room temperature before taking. Manipulate formamide in a chemical hood.
- 0.1× SSC: Add 5.97 mL of Nuclease-Free Water (Invitrogen, Waltham, MA, USA) to a 15 mL tube first and then add 30 µL of 20× SSC (Invitrogen, Waltham, MA, USA); mix the solution well. The buffer can be stored at room temperature for up to one week.

## 4. Experimental Procedures

### 4.1. Cell Culture

*Drosophila* S2 cells (male) (Thermo Fisher Scientific, Waltham, MA, USA), derived from late-stage 20–24 h-old *D. melanogaster* embryos, were cultured in Gibco Schneider’s *Drosophila* medium supplemented with 10% fetal bovine serum (SDM growth medium) (ExCell Bio, Shanghai, China) and maintained at 27 °C. *Drosophila* Kc167 cells (female), derived from *D. melanogaster* embryos, were cultured in SDM growth medium and maintained at 27 °C. GM12878 cells (Coriell Institute for Medical Research, Camden, NJ, USA), *Homo sapiens* lymphoblastoid cell line with EBV transformation (EBV-positive), were cultured in Gibco RPMI 1640 Medium (Gibco, Waltham, MA, USA) supplemented with 15% fetal bovine serum (ExCell Bio, Shanghai, China) and maintained at 37 °C with 5% CO_2_. RAMOS cells (ATCC, Manassas, VA, SA), *Homo sapiens* B lymphocytes cell line (EBV-negative), were cultured in Gibco RPMI 1640 (Gibco, Waltham, MA, USA) Medium supplemented with 10% fetal bovine serum (ExCell Bio, Shanghai, China) and 1% penicillin/streptomycin (Gibco, Waltham, MA, USA) and maintained at 37 °C with 5% CO_2_.

### 4.2. Probe Template Preparation

#### 4.2.1. Genomic DNA Extraction

Extract genomic DNA from 10 million freshly cultured cells according to the TIANamp Genomic DNA Kit manual. Quantify DNA concentration with Qubit referring to the manual.

PAUSE POINT: store genomic DNA aliquots at −20 °C for future use.

#### 4.2.2. Genomic Loci Amplification

Amplify genomic loci as probes template.

1. Design primers: Use the UCSC Genome Browser RepeatMasker track (https://genome.ucsc.edu/, accessed on 31 January 2024) to select non-repetitive regions as target genomic loci, perform primer design using Primer3 (https://primer3.ut.ee/, accessed on 31 January 2024), and further verify through BLAST analysis (https://blast.ncbi.nlm.nih.gov/Blast.cgi, accessed on 31 January 2024) to ensure that the amplified regions are unique and free from repetitive sequences.
2. Set up PCR mix in a PCR tube on ice (vol. 50 μL) and mix well: 25 µL of 2× Phanta Max Buffer, 1 µL of 10 mM dNTP, 2 µL of 10 µM Forward primer, 2 µL of 10 µM Reverse primer, 100 ng of genomic DNA, 1 µL of Phanta Max Super-Fidelity DNA Polymerase, and Nuclease-Free Water up to 50 µL.
3. Incubate in a thermal cycler (BioRad, Hercules, CA, USA) using the following program: Heat the lid at 105 °C, 1 cycle of initial denaturation for 3 min at 95 °C, 35 cycles of denaturation, annealing and extension at 95 °C for 15 s, 55 °C (adjust annealing temperature according to primers’ melting temperature) for 15 s, and 72 °C for several minutes (adjust extension time according to product length, the extension time for 1 kb typically ranges from 30 to 60 s) respectively, a final extension for 5 min at 72 °C, and hold at 4 °C.
4. Purify PCR product with DNA Clean & Concentrator-5 (Zymo Research, Irvine, CA, USA) and quantify DNA with Qubit (Invitrogen, Waltham, MA, USA) and Qsep100 (Bioptic, Beijing, China) referring to the manual.

PAUSE POINT: store probe template aliquots at −20 °C for future use.

### 4.3. Assembly of Tn5-Labeled Transposome 

CRITICAL: the procedure in step 4.3 should be carried out in the dark to protect from light.

#### 4.3.1. Adaptor A/B Annealing

Adaptor A is annealed through base pairing between oligo ME and oligo ME_A, while adaptor B is annealed through base pairing between oligo ME and oligo ME_B.

1. Add 1× TNE buffer to dissolve designed and synthesized oligos (ME, ME_A, and ME_B) to a concentration of 100 μM. Vortex the oligos for 10 s and then leave the solution for 12 h at 4 °C to ensure complete dissolve.

Annealing ratio testing:

2. Prepare 1:1.1 ratios of ME and ME_A/ME_B oligos by mixing 5 μL of ME with 5.5 μL of ME_A or 5 μL of ME with 5.5 μL of ME_B.
3. Run the reaction on the PCR machine using the following program: Heat the lid at 105 °C, incubate at 95 °C for 2 min; ramp from 95 °C to 75 °C with 0.1 °C/s, incubate at 75 °C for 2 min; ramp from 75 °C to 65 °C with 0.1 °C/s, incubate at 65 °C for 2 min; ramp from 65 °C to 50 °C with 0.1 °C/s, incubate at 50 °C for 2 min; ramp from 50 °C to 37 °C with 0.1 °C/s, incubate at 37 °C for 2 min; ramp from 37 °C to 20 °C with 0.1 °C/s, incubate at 20 °C for 2 min; ramp from 20 °C to 4 °C with 0.1 °C/s, incubate at 4 °C for 2 min; hold at 4 °C.
4. Dilute ME, ME_A, ME_B and the annealed Adaptor A/B ten times and quantify the concentration with Qubit referring to the manual.
5. Run 200 ng of each single-stranded oligo as control alongside 200 ng of annealed Adaptor A/B on the same 4–20% (*w*/*v*) TBE gel at 120 V for 60 min.
6. Transfer TBE gel to a box containing 50 mL of 1× TBE buffer and add 10 μL of Safe green to stain the DNA. Incubate the gel at room temperature for 15 min.
7. Image using imaging system Tanon 6100A under UV (Tanon, Shanghai, China).

Annealing scale up:

8. Choose the optimal ratio between ME and ME_A/ME_B oligos (i.e., only annealed strand is observed, with no detectable unannealed single oligo in the lane) and mix the rest of the ME and ME_A/ME_B oligo stocks with the optimal ratio and then run the annealing program. Divide the annealed Adaptor A/B into 50 μL of aliquots.

PAUSE POINT: store Adaptor A/B aliquots in the dark at −20 °C for future use.

#### 4.3.2. Tn5-Labeled Transposome Assembly

1. Tn5 transposase and Adaptor A/B are used to assemble the Tn5-labeled transposome (BGI, Shenzhen, China).
2. Take out Tn5 transposase (1 U/µL) from −80 °C and thaw on ice.
3. Set up Transposome Assembly Mix in 1.5 mL tube on ice and mix well: 4.8 µL of Adaptor A, 4.8 µL of Adaptor B, 120 µL of Coupling buffer, 16 µL of Tn5 transposase (1 U/µL) and 14.4 µL of Nuclease-Free Water (Invitrogen, Waltham, MA, USA).
4. Incubate the mixture for 1 h at room temperature.

PAUSE POINT: store Tn5-labeled transposome at −20 °C for future use.

### 4.4. Tn5-Labeled Transposome Fragmentation Testing

Proceed fragmentation testing to optimize the amount of Tn5-labeled transposome used in probes preparation. The greater the amount of Tn5-labeled transposome used, the shorter the fragments generated.

CRITICAL: the procedure in step 4.4 should be carried out in the dark to protect from light.

Tn5 fragmentation:

1. Prepare the reaction in a PCR tube on ice (vol. 50 μL), add Nuclease-Free Water in advance, and then add 50 ng of probes template DNA and 12.5 µL of 4× THS TD buffer, mix 10–20 times by pipetting with setting at 30 μL, briefly spin down, finally add Tn5-labeled transposome, mix 10–20 times by pipetting with setting at 30 μL, spin down briefly, and make sure there are no bubbles.
2. Incubate in a thermal cycler using the following program: heat the lid at 70 °C, incubate at 55 °C for 10 min, and hold at 4 °C.

Tn5 release, decrosslinking, and purification:

3. Add 50 μL of ChIP Elution buffer and 1 μL of Proteinase K to fragmented DNA (final concentration of SDS is 0.5%), mix, and incubate on Thermomixer (900 rpm) for 30 min at 65 °C.
4. Purify DNA with DNA Clean & Concentrator-5 and quantify with Qubit and Qsep100 referring to the manual.

Well-fragmented DNA can be used as FISH probes.

CRITICAL: The ratio between Tn5 and DNA is critical for optimal reactions, and this ratio needs to be empirically determined. Recommended testing amount of Tn5-labeled transposome for 50 ng DNA fragmentation is 8 µL. The majority of DNA fragments should fall into the 100–1000 bp range, with a peak at 150 bp.

PAUSE POINT: store FISH probe aliquots in the dark at −20 °C for future use.

### 4.5. FISH Probes Preparation

According to Tn5-labeled transposome fragmentation testing, choose optimal ratio between Tn5 and DNA and scale up fragmentation reactions. Procedure is the same as Tn5-labeled transposome fragmentation testing, and prepare enough FISH probes for DNA FISH experiment (typically use 5 ng probes for one DNA-FISH sample).

### 4.6. Tn5-Labeled DNA-FISH Experimental Protocol

Take *Drosophila* S2 cell DNA-FISH as an example.

#### 4.6.1. Coverslip Preparation

1. Take out one 6-well plate and put one coverslip in a well.
2. Wash with 1 mL of pure ethanol twice.
3. Dry in air at 37 °C for 1 h until completely dry.
4. Add l00 μL of Poly-L-Lysine onto coverslip.
5. Incubate for more than 20 min at room temperature.
6. Remove the Poly-L-Lysine and dry in air at room temperature for 1 h until completely dry.
7. Wash the coverslip with 2 mL of DPBS once.
8. Dry in air at room temperature for 1 h until completely dry.

#### 4.6.2. Attached Cells to Coverslip

1. Dilute fresh cultured S2 cells with 27 °C pre-warmed SDM growth medium to a concentration of 2.5 million cells/mL.
2. Add 2 mL of cells (5 million cells) onto the coverslip.
3. Incubated at 27 °C for 30 min.
4. Then, check the cell density under microscopy and wash with 27 °C pre-warmed DPBS twice.

#### 4.6.3. Cellular Fixation

CRITICAL: manipulate in a chemical hood.

1. Add 2 mL of 4% PFA onto the coverslip.
2. Incubate for 10 min at room temperature.
3. Then, wash with 2 mL of DPBS three times.

#### 4.6.4. Permeabilization

1. Add 2 mL of freshly prepared 0.5% Triton X-100/DPBS onto the coverslip.
2. Incubate for 5 min at room temperature.
3. Then, wash with 2 mL of DPBS once.

#### 4.6.5. RNase A Treatment

1. Add 2 mL of 100 µg/mL RNase A/DPBS onto the coverslip.
2. Incubate for 1 h at 37 °C.
3. Then, wash with 2 mL of DPBS three times.

#### 4.6.6. 20% Glycerol Treatment

1. Add 2 mL of 20% glycerol/DPBS onto the coverslip.
2. Incubate for 30 min at room temperature.

#### 4.6.7. Liquid Nitrogen Freeze

CRITICAL: beware of frostbite.

1. Freeze the coverslip in liquid nitrogen for approximately 30 s.
2. Then, thaw the frozen coverslip in air.
3. Once the frozen layer disappears, put the coverslip back into glycerol.
4. Repeat liquid nitrogen freeze once.

#### 4.6.8. HCl Denaturation

CRITICAL: manipulate in a chemical hood.

1. Remove the solution.
2. Add 2 mL of 0.1 M HCl onto the coverslip.
3. Incubate for 5 min at room temperature.
4. Then, wash with 2 mL of 2× SSC three times.

#### 4.6.9. Preparation for Hybridization

1. Rinse the coverslip with 2 mL of 2× SSC/50% formamide once and then remove the solution.
2. Add 2 mL of fresh 2× SSC/50% formamide.
3. Incubate for 30 min at room temperature.

#### 4.6.10. Hybridization

CRITICAL: from now on, the following procedure should be protected from light.

Humidified chamber preparation:

1. Prepare a 15 cm plate, put paper towels in the plate and wet them with 40 mL of 2× SSC, and pre-warm at 42 °C.

Probes denaturation:

CRITICAL: 110 µL of hybridization buffer with probes is to be used for 25 mm diameter coverslip. Do not pre-warm the parafilm for too long; limit it to no more than 10 min, as the parafilm may melt.

2. Set up one tube of 5 ng of FISH probes in 110 µL of hybridization buffer in a PCR tube (Sangon Biotech, Shanghai, China).
3. Denature the hybridization buffer with probes for 10 min at 80 °C in a PCR machine.
4. At the same time, pre-warm a piece of ~7 × 7 cm^2^ parafilm on the 80 °C magnetic stirrer (CRYSTAL, Shanghai, China).

Denature coverslip with probes:

5. Quickly add 110 µL of hybridization buffer with probes (pre-denatured at 80 °C) onto the center of the parafilm on the 80 °C magnetic stirrer.
6. Take out the coverslip and turn it cell-side down onto the hybridization buffer with probes.
7. Denature the coverslip with probes for 5 min at 80 °C.

Hybridization overnight:

8. Transfer the slide with parafilm onto the wet paper towels in the humidified chamber and incubate at 42 °C for 5 min.
9. Then, add 20 mL of H_2_O to the surroundings of paper towels and seal the plate surroundings with parafilm.
10. Incubate the slide in dark humidified chamber at 42 °C overnight.

Post-hybridization washing:

11. Take out the coverslip and then turn it cell-side up onto the original 6-well plate containing 2 mL of 2× SSC/50% formamide.
12. Wash three times with 2 mL of 2× SSC at 42 °C for 5 min each.
13. Wash three times with 2 mL of 0.1× SSC at 42 °C for 5 min each.
14. Wash twice with 2 mL of DPBS at room temperature for 5 min each.
15. Add 2 mL of DPBS and keep at 4 °C before microscopy observation.

#### 4.6.11. Microscopy and Analysis

After completing the hybridization and washing steps, mount the slides with an appropriate antifade mounting medium to preserve fluorescence signals. Use a fluorescence microscope equipped with suitable filter sets to detect the fluorophores used in the probes. High-resolution imaging techniques, such as confocal microscopy or structured illumination microscopy (SIM), are recommended to capture detailed spatial information of the labeled loci. Acquire z-stack images to ensure precise three-dimensional localization of the signals within the nucleus.

For quantitative analysis, process the acquired images using image analysis software (e.g., ImageJ (Version 1.53t, Wayne Rasband, National Institutes of Health, Bethesda, MD, USA, https://imagej.nih.gov/ij/, accessed on 31 January 2024), Fiji (Version 2.14.0, community-driven project, https://fiji.sc/, accessed on 31 January 2024), or Imaris (Version 10.0, Bitplane, Zurich, Switzerland, https://imaris.oxinst.com/, accessed on 31 January 2024)) to identify and quantify fluorescence signals. The spatial proximity of labeled loci can be analyzed by measuring the distances between signals in three-dimensional space. For statistical evaluation, calculate the frequency of co-localization or proximity between loci across multiple nuclei. Ensure proper background subtraction and signal thresholding to minimize noise and improve the accuracy of the analysis.

## 5. Results

### 5.1. Amplification of Target Genomic Regions as Templates for FISH Probes

**Figure 2:**
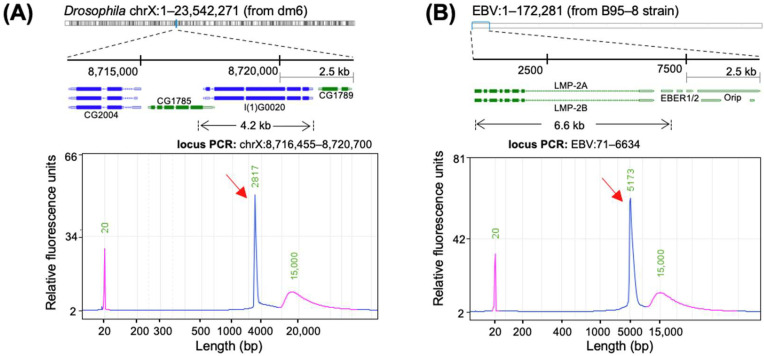
Amplification of target genomic loci. (**A**) PCR amplification of the target locus *l(1)G0020* on the *Drosophila* X chromosome using S2 genomic DNA as the template. (**B**) PCR amplification of the target locus from the EBV genome (B95-8 strain) using GM12878 genomic DNA containing the EBV genome as the template. In both (**A**,**B**), the top bars represent the chromosome, the dashed line indicates the region of interest, blue bars represent negative-strand genes, and green bars represent positive-strand genes, the black arrows indicate the target amplification regions. Capillary electrophoresis confirms the presence of single, specific amplicons (red arrows), with the purple peak representing the marker.

We successfully amplified the *l(1)G0020* gene located on the X chromosome of *Drosophila* S2 cells and an EBV fragment from the B95-8 EBV genome. Genomic DNA was first extracted from *Drosophila* S2 cells and GM12878 cells and used as templates for PCR. Specific primers were designed to target the *l(1)G0020* gene (~4 kb) (Figure 2A) and the EBV fragment (~6 kb) (Figure 2B). The PCR products were analyzed using the Qsep100 nucleic acid capillary electrophoresis system, as shown in Figure 2A,B. The results confirmed that the amplification products were singular, with fragment lengths matching the target regions. This demonstrates that the method efficiently and specifically amplified the target templates, making them suitable for FISH probe preparation.

### 5.2. Synthesis and Fluorescent Labeling of Oligos for Tn5-Labeled Transposome Assembly

We synthesized oligos for Cy5.5 fluorescent labeling. Based on the characteristics of the Tn5 transposase, we designed and synthesized the oligos ME, ME_A, and ME. Through annealing, ME was paired with ME_A and ME, respectively, to form double-stranded structures referred to as Adaptor A and Adaptor B.

**Figure 3:**
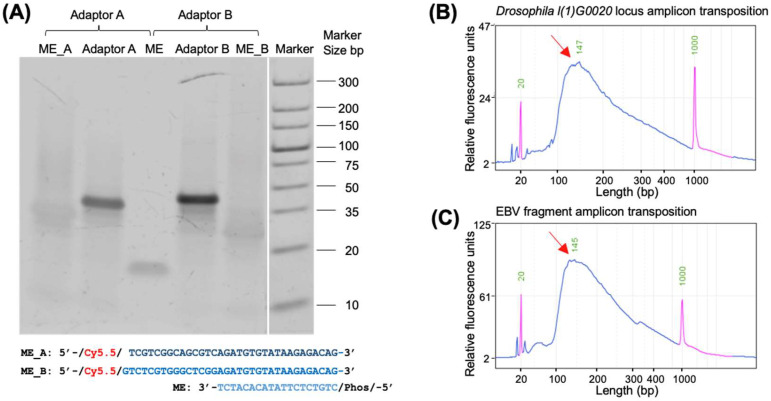
Fluorescently labeled adaptor synthesis, assembly, and probe size determination. (**A**) PAGE gel analysis of Adaptor A (ME and ME_A) and Adaptor B (ME and ME_B) annealing. The Cy5.5 fluorophore is shown in red. ME_A (dark blue) or ME_B (blue) is annealed with ME (light blue) to form Adaptor A and Adaptor B, respectively. The annealing of ME and ME_A/ME_B oligos at a molar ratio of 1:1.1 resulted in slower migration and distinct, specific bands, indicating successful annealing with the correct proportions. The white line indicated a splicing between ME_B lane and marker lane. (**B**) DNA fragment size distribution after Tn5-labeled transposome-mediated fragmentation of the *Drosophila l*(*1*)*G0020* locus amplicon. (**C**) DNA fragment size distribution after Tn5-labeled transposome-mediated fragmentation of the EBV fragment amplicon. In both (**B**,**C**), the DNA fragments are predominantly around 150 bp, with red arrows indicating the peak positions and the purple peak representing the marker.

Optimizing the molar ratio of ME and ME_A/ME_B oligos for annealing was critical for improving the specificity of subsequent DNA-FISH. Excess oligos could result in fluorescently labeled ME_A/ME_B oligos contributing to background noise; therefore, the molar ratio was carefully adjusted. When the molar ratio was set to 1:1.1, electrophoresis, as shown in Figure 3A, revealed that the molecular weight of the bands in the adaptor (Adaptor A/B) lanes after annealing was significantly higher than that of the oligo bands before annealing. Additionally, no nonspecific bands were observed below the main bands.

These results demonstrated that the optimized annealing ratio and program were highly suitable for the annealing of Tn5-labeled adaptors, allowing the oligos of both strands of the adaptor to anneal effectively without residual oligos. Adaptor A and Adaptor B were then assembled with Tn5 to form Tn5-labeled transposome for subsequent use. Fully annealed adaptors provided a reliable foundation for the assembly of Tn5 and significantly enhanced the specificity of DNA-FISH.

### 5.3. Preparation of FISH Probes Using Tn5-Labeled Transposome

To prepare FISH probes, the amplified *l(1)G0020* gene and EBV fragment were used as input DNA, while the assembled Tn5-labeled transposome was utilized to fragment the templates. During this process, the Tn5 transposase simultaneously fragmented the DNA and transposed fluorescently labeled adaptors onto the target sequences, resulting in the generation of fluorescence-labeled probes. This dual functionality of Tn5 ensured efficient fragmentation and labeling in a single step, streamlining the probe preparation process.

The Tn5-transposed fragments were analyzed using electrophoresis, as shown in Figure 3B,C. The results demonstrated that the templates were efficiently fragmented into diffuse fragments ranging from 20 to 1000 bp, with a peak at approximately 150 bp. The uniform distribution of fragment sizes and the absence of large, unfragmented DNA indicated the high efficiency of the Tn5 transposase in processing the input DNA.

The Tn5-generated probes offered several advantages. The smaller target regions and shorter fragments provided higher resolution, enabling more precise localization of target sequences during DNA-FISH. Additionally, the streamlined preparation process reduced the time and complexity associated with probe generation, making this method more efficient and scalable for various applications. These results highlight the suitability of Tn5-labeled transposome-based probes for high-resolution DNA-FISH experiments.

### 5.4. Image Analysis of Tn5-Labeled DNA-FISH

We evaluated the specificity and performance of Tn5-labeled DNA-FISH for imaging both endogenous and exogenous genomic loci.

**Figure 4:**
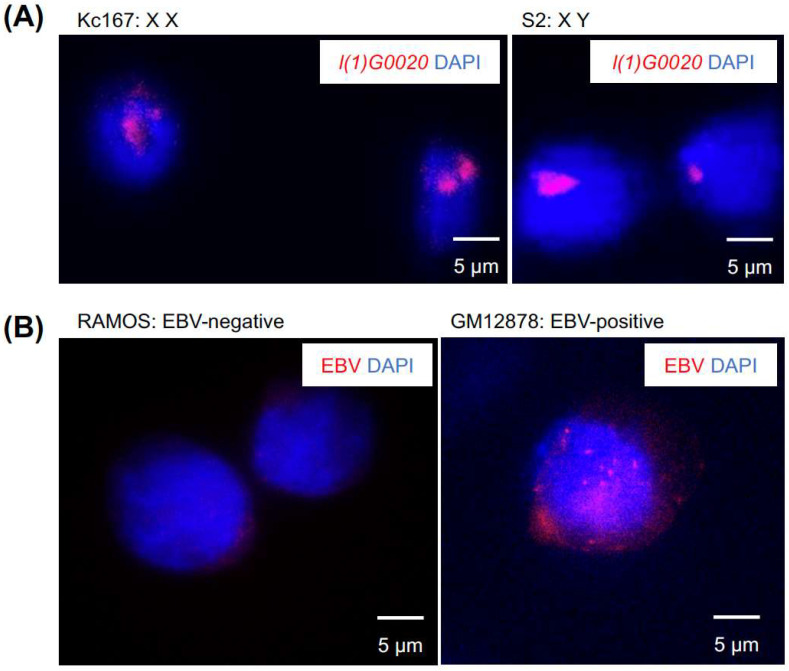
Application of Tn5-labeled DNA-FISH for imaging endogenous and exogenous genomic loci. (**A**) Tn5-labeled probes targeting the *Drosophila* X chromosome *l(1)G0020* locus were hybridized with Kc167 cells (female with two X chromosomes) and S2 cells (male with one X chromosome). DNA-FISH results show two foci in Kc167 cells and one focus in S2 cells, consistent with the localization of the target region on the X chromosome. Probes for *l(1)G0020* are labeled in red (Cy5.5), and nuclei are stained with DAPI (blue). (**B**) Tn5-labeled probes targeting the EBV genome were hybridized with RAMOS cells (EBV-negative) and GM12878 cells (EBV-positive). DNA-FISH results show multiple EBV episomes in GM12878 cells, while no fluorescence signal is detected in RAMOS cells, demonstrating high probe specificity. Probes for the EBV genome are labeled in red (Cy5.5), and nuclei are stained with DAPI (blue).

For endogenous loci imaging, we targeted the *l(1)G0020* gene locus located on the X chromosome in *Drosophila* cells. DNA-FISH experiments were performed using probes prepared with the Tn5-labeled transposome on *Drosophila* Kc167 (female, XX) and S2 (male, XY) cells. The imaging results (Figure 4A) showed distinct and specific foci corresponding to the expected chromosomal configurations. In female Kc167 cells (XX), two bright foci were observed, consistent with the presence of two X chromosomes. In male S2 cells (XY), a single, larger focus was detected, reflecting the single X chromosome in these cells. These results confirm the ability of Tn5-labeled probes to specifically detect endogenous loci with high accuracy.

For exogenous loci imaging, we targeted the EBV genome in human cells. DNA-FISH was conducted on RAMOS (EBV-negative) [19] and GM12878 (EBV-positive) cells [20] using probes prepared with the Tn5-labeled transposome. The imaging results (Figure 4B) demonstrated clear specificity: no foci were observed in EBV-negative RAMOS cells, while multiple distinct foci were detected in EBV-positive GM12878 cells, corresponding to the integrated EBV genome.

The high specificity and accuracy of Tn5-labeled DNA-FISH were evident in both endogenous and exogenous loci imaging. This method successfully distinguishes between the presence and absence of the EBV genome, with potential applications for identifying different chromosomal configurations and genomic states within nuclei. Additionally, the brightness and clarity of the foci in male and female *Drosophila* nuclei further highlight the effectiveness of Tn5-labeled probes, providing solid evidence for high-resolution genomic imaging [18]. These results validate the robustness and versatility of the Tn5-labeled DNA-FISH approach for detecting both native and foreign genomic loci.

## 6. Conclusions

This study presents Tn5-labeled DNA-FISH as a simplified and cost-effective method for probe preparation for chromatin imaging. By harnessing the dual functionality of the Tn5 transposase and fluorescently labeled adaptors [21], this approach integrates DNA fragmentation and labeling into a single streamlined step, significantly simplifying the workflow. The resulting probes, with fragment sizes averaging ~150 bp and spanning regions as small as 4–6 kb, provide superior resolution and high-fluorescence signal density.

Tn5-labeled DNA-FISH demonstrated exceptional specificity and performance in detecting both endogenous and exogenous genomic loci, as evidenced by its ability to resolve chromosomal configurations in *Drosophila* cells and distinguish the presence or absence of the EBV genome in human cells. By enabling precise localization of genomic regions at the kilobase scale, Tn5-labeled DNA-FISH provides a powerful and accessible tool for probe generation, facilitating the study of genome architecture, chromatin spatial organization, and gene regulation.

## Figures and Tables

**Table 1:** Oligo sequences and synthesis purification methods.

Oligo ID	Sequence (5′-3′)	Purification ^1^
Oligos for adaptor annealing		
ME	/5Phos/CTGTCTCTTATACACATCT	HPLC
ME_A	/5Cy5.5/TCGTCGGCAGCGTCAGATGTGTATAAGAGACAG	HPLC
ME_B	/5Cy5.5/GTCTCGTGGGCTCGGAGATGTGTATAAGAGACAG	HPLC
Primers for *Drosophila l(1)G0020* gene amplification		
Forward primer	TTCTCCAGGAATCCCAGATG	DSL
Reverse primer	CGTAAATCTGCCCGAGGATA	DSL
Primers for EBV genome one fragment amplification		
Forward primer	TGCCTGCCTGTAATTGTTGC	DSL
Reverse primer	GGTCCTGAGGTTTTGCAGTG	DSL

^1^ Oligo synthesis purification methods: HPLC (high-performance liquid chromatography); DSL (desalting).

## Data Availability

The original contributions presented in this study are included in the article. Further inquiries can be directed to the corresponding authors.
